# Editorial: Advances in viromics: new tools, challenges, and data towards characterizing human and environmental viromes

**DOI:** 10.3389/fmicb.2023.1290062

**Published:** 2023-09-26

**Authors:** Mária Džunková, Cristina Moraru, Karthik Anantharaman

**Affiliations:** ^1^Institute for Integrative Systems Biology, University of Valencia and Consejo Superior de Investigaciones Científicas (CSIC), Valencia, Spain; ^2^Environmental Metagenomics, Faculty of Chemistry, Research Center One Health Ruhr of the University Alliance Ruhr, University of Duisburg-Essen, Essen, Germany; ^3^Department of Bacteriology, University of Wisconsin-Madison, Madison, WI, United States

**Keywords:** bacteriophage, viromics, computational tools and databases, taxonomy, freshwater ecosystem

Bacteriophages (viruses of bacteria) are incredibly diverse. They can either reduce the population of their bacterial host, or carry on infection without causing the cell lysis, which can be even beneficial to their host (Chevallereau et al., 2022). Studying bacteriophages is important for understanding how they regulate bacterial populations, which can have practical applications in healthcare, agriculture, biotechnology, and environmental management (Domingo-Calap et al., 2016; Ye et al., 2019). While most of the knowledge on bacteriophage biology has been gathered from experiments with cultures of phages in laboratories, the largest diversity of bacteriophages has been revealed by DNA sequencing of environmental samples, in which most of the bacteria are yet-to-be-discovered and yet-to-be-cultured (Bodor et al., 2020). Bacteria harbor universal marker genes which help to estimate their diversity and target environments with a large proportion of novel bacterial taxa, however, this is not the case of the bacteriophages (Low et al., 2019). The lack of a universal marker gene has been hindering bacteriophage research in the beginning of the viromics era, nevertheless, fortunate advancements in this field in the recent years have been achieved by a multitude of emerging computational tools.

The expansion of the number of computational tools for analysis of viromes is triggered by an urgent need to improve detection of novel types of bacteriophages, to predict functions of phages containing large portion of novel proteins, and to adapt the taxonomic classification system to the large number of emerging phages (Andrade-Martínez et al., 2022). Thanks to these computational efforts, researchers can conduct comprehensive studies on how bacteriophages influence various biological aspects in diverse environments without the need to culture the phages. However, the effectiveness of the current computational tools is affected by various factors, including distinct training sets of samples utilized during their development, diverse reference sequence databases, and variations in how they handle bacterial sequence contamination. The objective of the Research Topic “*Advances in viromics: new tools, challenges, and data toward characterizing human and environmental viromes*” is to address the emerging challenges in the field of viromics.

The most challenging issue in the computational analysis of viromes is choosing the best tool for the detection and selection of phage contigs. The study of Schackart et al. evaluates the performance of five homology-based tools: VirSorter (Roux et al., 2015), MARVEL (Amgarten et al., 2018), viralVerify (Antipov et al., 2020), VIBRANT (Kieft et al., 2020), and VirSorter2 (Guo et al., 2021), and three sequence-based tools: VirFinder (Ren et al., 2017), DeepVirFinder (Ren et al., 2020), Seeker (Auslander et al., 2020). The initial study design involved 19 tools, however, 11 of them were discarded due to runtime exceptions, hard-coded paths, lack of clear documentation, scalability issues, and inability to run instances on different cores, which are the common reasons why many bioinformatic tools are not widely used by the scientific community. The eight remaining tools were assessed in terms of their precision, sensitivity and specificity. The effects of contig length, phage taxonomy, sequencing and assembly errors, eukaryotic contamination, low viral content, and sequencing error were examined. Their performance was evaluated using four benchmark datasets: (1) set of fragments from complete genomes, (2) phageome set created from simulated reads from phage genomes and then assembled and binned, (3) simulated metagenome set created the same way as the phageome, but with marine samples, (4) colorectal cancer and gut virome datasets coming from real metagenomes.

All tools, especially the homology-based tools, showed reduced sensitivity for non-caudovirales sequences. Similarly, the low viral abundance in simulated metagenomes decreased precision of all tools, although the homology-based tools performed slightly better and they were also more robust to eukaryotic contamination. In contrast, the sequence-based tools performed better with shorter contigs. It is important to note that in the case of real metagenomes (non-simulated data), there was only a little overlap between the eight tested tools. The results suggest that the choice of tools depends on the specific research question and the type of metagenomes being analyzed. Schackart et al. recommend using DeepVirFinder for analysis of purified viromes due to its high sensitivity. DeepVirFinder and Metaphinder were shown to be very efficient in identification of novel phages. For studying dominant phages with a high confidence, VirSorter2 or viralVerify are suggested. In summary, a multi-tool approach combining sequence-based and homology-based tools is recommended. It is also important to keep in mind that other aspects of performance, such as parameter tuning, database update, and ability to detect prophage and plasmid sequences might be considered in decision making when selecting the most suitable computational tool for virome analysis. We believe that conducting similar studies on a regular basis will be very useful for keeping up to date with the emerging computational tools, and constant updates of the phage sequence databases and phage taxonomy.

The importance of automatic updates in the rapidly expanding field of viromics is underlined in the report of Albrycht et al.. This team developed Phage & Host Daily (PHD) web application (http://phdaily.info) that accesses reliable information on phage host specificity by publishing daily reports on phage-host interactions. The information is extracted from three large databases: International Committee on Taxonomy of Viruses (ICTV, Krupovic et al., 2021), National Center for Biotechnology Information (NCBI, Sayers et al., 2019) and the Genome Taxonomy Database (GTDB, Parks et al., 2022). The website allows users to search for specific taxa or browse taxonomic trees to access information on virus-host interactions. The results are presented as a table of pairwise interactions with details on source databases, taxonomic affiliations, genome composition, and assembly completeness. Additionally, PHD supports the construction of custom datasets for machine learning algorithms, including viruses from metagenomic sequences. The continuous updates of PHD align with taxonomic changes, contributing to a comprehensive classification scheme for phage research.

Viral taxonomy has undergone major transformations recently, with the Baltimore Classification being replaced by the “Megataxonomy of Viruses” (Koonin et al., 2020), in which viruses are grouped into 6 different realms, based on their evolutionary history. Within each realm, there are further 10 taxonomic levels comprising the scaffold for the hierarchical classification of viruses. In the case of phages, the most dramatic taxonomic changes affected the tailed dsDNA phages. These were all grouped until recently under the orphan *Caudovirales* order, into three families (*Podoviridae, Myoviridae*, and *Syphoviridae*). Under the new taxonomic system, the *Caudovirales* order and the three families were dissolved (Adriaenssens et al., 2021), because accumulated evidence suggested that they are not monophyletic. All tailed dsDNA phages are now classified in the *Duplodnaviria* realm, in the *Caudoviricetes* class. Their classification at the order and family level is currently in progress, and this has increased the need for dedicated bioinformatics tools. In their review, Zhu et al. compared the performance of different tools to assign viral contigs to existing taxons, from family to genus level. The four tools that passed the initial classification criteria (e.g., having updatable databases) were CAT, PhaGCN, MMseq2, and vConTACT 2.0. These were further evaluated using a variety of metrics and several test datasets. Each tool showed both strengths and weaknesses. For example, some tools showed higher accuracy but underperformed in the case of short contigs. Again, the choice of tools to use is in the hands of the researcher performing the study, depending on the scientific questions.

Large sequencing efforts in the last decades have revealed an enormous genetic diversity of bacteriophages and shed light on their ecological roles. Viruses in aquatic systems have received significant attention, but much of these efforts have been focused on marine systems (Coutinho et al., 2017; Breitbart et al., 2018; Ignacio-Espinoza et al., 2019). Consequently, freshwater viruses have remained significantly underexplored. To generate a comprehensive collection of freshwater viral genomes, Elbehery and Deng studied viruses from twelve representative freshwater environments (biomes) ranging from lakes, estuaries, wastewater, groundwater, and applied environments such as ballasts and fishponds. Publicly available metagenomic data from these environments was downloaded and quality control was conducted to ensure the removal of cellular contamination. This was followed by identification of viruses, establishment of viral clusters, prediction of hosts for viruses, and identification of viral auxiliary metabolic genes (AMGs). Overall, the study expands the known diversity of freshwater viruses and sheds light on their ecology in diverse freshwater biomes. Amongst the freshwater biomes studied, the authors found significant variance in viral diversity between biomes, and also with samples from the same biome. While it is possible that some of these findings may be attributed to insufficient sequencing depth of viruses in these samples, the authors argue that viral diversity is dictated by changing environmental chemistry as posited previously (Adriaenssens et al., 2021) and also suggest a limited complexity of the freshwater core virome as observed in other environments such as the human gut and the oceans which are dominated by double stranded DNA viruses (now *Duplodnaviria* realm) in the class *Caudoviricetes* (Brum et al., 2015; Broecker et al., 2017). Finally, the study identifies a significant complement of AMGs in freshwater viruses. AMGs are host-derived genes on viral genomes that can provide increased fitness and infection efficiency to viruses. Freshwater AMGs identified include genes for degradation of aromatic and xenobiotic compounds and photosynthesis. Overall, Elbehery and Deng provide a collection of viral genomes that can serve as a foundation to investigate the ecology and evolution of freshwater viruses. Future studies can build upon this groundwork to focus on extraction of viromes (purified virus particles), deeper sequencing of freshwater viruses, and a holistic interpretation of freshwater viruses in the context of chemistry and their hosts to understand their roles, dynamics, and importance in freshwater systems.

The last study in this Research Topic highlights the differences between the traditional culture methods and different types of viromics datasets (dsDNA, ssDNA, dsRNA, and ssRNA) by testing wastewater treatment plant samples against strains of *Brevundimonas* and *Serratia*. In this study, Friedrich et al. associated the environmental viruses with these two bacterial host species by sequencing the phage particles harvested after the initial overlay and compared the resulting community to the collection of viruses obtained by traditional plaque assays. It was found that the majority of the dominant phages were successfully isolated, but a considerable diversity remained unavailable, including phages closely related to the phages isolated by the traditional plaque assay. Interestingly, this study provided experimental evidence for the first phage with a class-level host range, vB_SmaM-Otaku, which was found to infect both tested host strains belonging to two different classes of *Pseudomonadota*. Previous computational studies suggested existence of phages with a very broad host range (Paez-Espino et al., 2016), however, this is the first time it has been experimentally confirmed. The study also emphasizes the need for novel approaches to explore the unexplored viral diversity, especially ssDNA and RNA phages due to their unique characteristics and smaller genome sizes compared to DNA phages.

We can anticipate that in the very near future emerging direct RNA-Seq technologies, long-read sequencing, new whole genome amplification methods and novel experimental methods for linking unculturable phages to their hosts will generate a large amount of knowledge and sequence data (Smith et al., 2022). It is expected that as our knowledge on bacteriophages expands, a corresponding increase in challenges is likely to arise. The recent research efforts have shown that combining DNA sequencing with experimental methods can provide evidence-based discoveries revealing how phages operate within a specific environment, e.g., by highlighting the importance of metabolic auxiliary genes in sulfur ecology (Kieft et al., 2021), or the role of phage-plasmids in spread of antibiotic resistances (Pfeifer et al., 2022). In light of these evolving technologies and innovative research approaches, it is clear that our understanding of phage biology will continue to expand.

## Author contributions

MD: Writing—original draft, Writing—review and editing. CM: Writing—original draft, Writing—review and editing. KA: Writing—original draft, Writing—review and editing.
